# Oxidative stress response signaling pathways in trabecular meshwork cells and their effects on cell viability

**Published:** 2013-06-12

**Authors:** Nanako Awai-Kasaoka, Toshihiro Inoue, Takanori Kameda, Tomokazu Fujimoto, Miyuki Inoue-Mochita, Hidenobu Tanihara

**Affiliations:** 1 Department of Ophthalmology, Faculty of Life Sciences, Kumamoto University, Kumamoto, Japan; 2 Department of Ophthalmology and Visual Sciences, Kyoto University Graduate School of Medicine, Kyoto, Japan

## Abstract

**Purpose:**

To clarify the primary oxidative stress response signaling pathways in trabecular meshwork (TM) cells and their effects on cell viability.

**Methods:**

Porcine TM cells were treated with 600 μM or 800 μM H_2_O_2_, and their time-dependent morphologic changes were observed. Phosphorylation of protein kinase B (Akt), extracellular regulated kinase (ERK)1/2, p38, and c-Jun NH2-terminal kinase (JNK) was evaluated by western blot analysis. The intracellular localization of NFκB was evaluated by western blot analysis. One-hour pretreatments with LY294002, U0126, and SB203580, with the inhibitors of PI3K, ERK1/2, and p38, respectively, were conducted to evaluate the roles of these molecules in the cellular reaction against H_2_O_2_. Cell viability was assessed using propidium iodide and anticleaved caspase-3 antibody.

**Results:**

TM cells treated with 600 μM H_2_O_2_ showed morphologic changes at 2 h that were partially recovered at 8 h after treatment. TM cells treated with 800 μM H_2_O_2_ did not recover, and the viability was significantly decreased. Both doses of H_2_O_2_ activated Akt, ERK1/2, and p38 in TM cells at 20 min after treatment, but not JNK or NFкB until 1 h after treatment. Inhibitors of PI3K, ERK1/2, and p38 suppressed recovery from the morphologic changes induced by 600 μM H_2_O_2_. Of these three inhibitors, the PI3K and ERK1/2 inhibitors decreased TM cell viability under oxidative stress.

**Conclusions:**

In TM cells, the PI3K-Akt, ERK, and p38 signaling pathways are primary oxidative stress response pathways involved in the mechanism of recovery from cellular morphologic changes induced by H_2_O_2_ treatment accompanied by actin cytoskeletal changes.

## Introduction

Intraocular pressure is determined by the balance between the inflow and outflow of the aqueous humor. Higher intraocular pressure is a significant risk factor for the progression of glaucoma, and is currently the only target for clinical therapeutic modalities [1-3]. The outflow pathway through the trabecular meshwork (TM) and Schlemm’s canal are the main pathways in humans [4-6], and the outflow facility of the pathways is decreased in eyes with glaucoma [7]. An underlying mechanism of decreased outflow is the overdeposition of extracellular matrix (ECM) in the outflow tissues [8]. TM cells are considered to regulate the amount of ECM, because they can simultaneously produce and degrade ECM with matrix metalloproteinases [8]. Thus, TM cell dysfunction might lead to deregulation of the essential turnover of ECM in outflow tissues, resulting in increased outflow resistance. Consistent with this hypothesis, the number of TM cells is decreased in glaucomatous eyes [7].

Oxidative stress is an important biologic phenomenon, and is well known to be involved in pathologies of many age-related diseases. Glaucoma is also an age-related disease, and oxidative stress has an important role in glaucoma pathology. For example, oxidative stress marker levels are significantly increased in the aqueous humor of glaucoma patients [9-11], suggesting that outflow tissues, including the TM, in glaucomatous eyes are continuously exposed to oxidative stress. In addition, oxidative DNA damage is significantly increased in the TM of glaucoma patients [12,13]. These findings indicate that oxidative damage occurs in the TM of glaucomatous eyes, and may abolish or reduce the function of the TM cells, leading to increased outflow resistance and the risk of glaucoma progression. Though proteolytic cellular systems are reported to have important roles in the oxidative stress response in TM cells [14], and chronic oxidative stress induces the activation of NFκB and the upregulation of proinflammatory markers [15], the intracellular signaling that is activated directly by oxidative stress has remained unclear. The purpose of this study was to investigate the signaling pathways directly involved in responding to oxidative stress in TM cells, and their effects on cell viability.

## Methods

### Trabecular meshwork cell culture and treatments

Porcine TM (PTM) cells were isolated from freshly obtained eyes (from a local abattoir) by collagenase digestion, and cultured as described previously [16]. Briefly, the lens, vitreous, iris, and ciliary body were removed from the anterior segments of porcine eyes, and the TM was scraped from the sclera. Isolated TM was digested using 1 mg/ml collagenase type 4 for 2 h, and then the tissue samples were centrifuged (270 ×g for 10 min), suspended in cell-culture medium, and plated on 2% gelatin-coated plastic dishes. TM cells were cultured in Dulbecco’s modified Eagle’s medium (DMEM; Wako Pure Chemical Industries, Osaka, Japan) supplemented with 10% heat-inactivated fetal bovine serum (HyClone Laboratories, Logan, UT) and antibiotics at 37 °C under 5% CO_2_. PTM cells were used at passage 5. To examine the effects of oxidative stress, cultured PTM cells were treated with 600 μM or 800 μM H_2_O_2_ at 37 °C after overnight serum starvation, and the time-dependent morphologic changes of the cells were observed under a microscope. When needed, LY294002 (10 μM, an inhibitor of PI3K; Calbiochem, Darmstadt, Germany), Akt inhibitor IV (Calbiochem), U0126, (10 μM, an inhibitor of ERK1/2; Cell Signaling Technology, Danvers, MA), and SB 203580 (10 μM, an inhibitor of p38; Sigma, St. Louis, MO) were also added to the medium 1 h before H_2_O_2_ treatment.

### Time-lapse imaging

Time-lapse imaging for PTM cells treated with 600 μM H_2_O_2_ was performed using an inverted microscope (Nikon Bio Station; Nikon, Tokyo, Japan) with a 20× objective lens (N.A. 0.8). The stage incubator and objective lens were kept at 37 °C, 5% CO_2_, and controlled humidity of 95% or greater. Time-lapse images were captured every 15 min using a CCD camera (DS-2MWc; Nikon) and analyzed using the NIS-Element Advanced Research imaging software (Nikon).

### Hoechst, propidium iodide staining

To distinguish living cells from dead cells, TM cells treated with H_2_O_2_ at 600 μM or 800 μM after overnight serum starvation were stained with Hoechst 33342 (Molecular Probes, Eugene, OR) and propidium iodide (PI; Molecular Probes). Hoechst 33342 and PI were added to the culture medium at 1 μg/ml for 30 min in TM cells treated with 600 μM or 800 μM H_2_O_2_, and TM cells stained with Hoechst 33342 and PI were observed by microscope; then the number of positive cells for each dye was counted. Hoechst 33342 enters both living and dead cells and stains their nuclei. PI is a membrane-impermeant dye for living cells, and only stains nuclei of dead cells.

### Immunocytochemistry

PTM cells were fixed with 4% paraformaldehyde in PBS for 15 min, and washed with cytoskeletal buffer (10 mM 2-morpholinoethansulfonic acid potassium salt, 150 mM NaCl, 5 mM EGTA, 5 mM glucose, pH 6.1) and serum buffer (10% FBS and 0.2 mg/ml sodium azide in PBS). The cells were permeabilized with 0.5% Triton X-100 in PBS for 12 min at room temperature and blocked with serum buffer at room temperature for 30 min. After blocking, the cells were incubated overnight at 4 °C with the rabbit anticleaved caspase-3 (Asp175) antibody (1:400, Cell Signaling Technology). The cells were rinsed with serum buffer and then incubated with antirabbit IgG secondary antibody Alexa Fluor 488 (Invitrogen, Carlsbad, CA) and Phalloidin-TRITC (Sigma-Aldrich, St. Louis, MO) for F-actin staining at room temperature for 30 min. After cells were washed with PBS, they were mounted with mounting medium with 4’,6-diamidino-2-phenylindole (VECTASHIELD; Vector Laboratories, Burlingame, CA) and observed using a fluorescence microscope (BX51; Olympus, Tokyo, Japan).

### Western blot analysis

The cell medium was removed, and the cells were washed three times with cold phosphate-buffered salt solution (PBS) and lysed with RIPA buffer (25 mM Tris-HCl [pH 7.6], 150 mM NaCl, 1% NP-40, 1% sodium deoxycholate, and 0.1% sodium dodecyl sulfate) supplemented with a protease inhibitor cocktail, ethylenediaminetetraacetic acid (EDTA) solution (Thermo Scientific), and Phosphatase Inhibitor Cocktail (Nacalai Tesque, Kyoto, Japan). Cytosol and nucleus fractions were extracted from TM cells using a ProteoExtract® Subcellular Proteome Extraction Kit (Calbiochem) according to the manufacturer’s protocol. After centrifugation, total protein in the supernatant was measured using a BCA™ Protein Assay Kit (Pierce, Rockford, IL) according to the manufacturer’s protocol. Equal amounts of protein (5 μg, except NFκB [9 μg]) were electrophoresed on a NuPAGE 10% Bis-Tris gel (Invitrogen). The proteins were transferred onto an Amersham Hybond-P polyvinylidene fluoride transfer membrane (GE Healthcare, Little Chalfont, UK) and blocked in 2% fat-free milk for 1 h at room temperature. The membranes were washed three times (10 min per wash) in Tris-buffered saline containing 0.1% Tween-20 (TBST), and incubated overnight at 4 °C with the following antibodies (Cell Signaling Technology) diluted in 5% BSA (MP Biomedicals LLC, Solon, OH): rabbit polyclonal antibody directed against phospho-Akt (Ser473; 1:1000), Akt (1:1000), phospho-ERK1/2 (1:1000), ERK1/2 (1:1000), phospho-p38 (1:1000), p38 (1:1000), phospho-JNK (1:1000), JNK (1:1000), and NFκB (1:1000) after 20, 40, and 60 min of H_2_O_2_ treatment. The membranes were washed three times (10 min per wash) in TBST and incubated with antirabbit or antimouse IgG conjugated with horseradish peroxidase (1:3000 in TBST; GE Healthcare) at room temperature for 1 h. Immunoreactive bands were visualized with an enhanced chemiluminescence detection kit (GE Healthcare).

### Statistical analysis

The intensities of protein bands in western blots were quantified using Image J (National Institutes of Health, Bethesda, MD). Student’s two-tailed test and Dunnett’s test were used to determine the statistical significance of differences between the control group and other groups. A p value of less than 0.05 was considered statistically significant.

## Results

### Effects of H_2_O_2_ on PTM cell morphology

**Figure 1:**
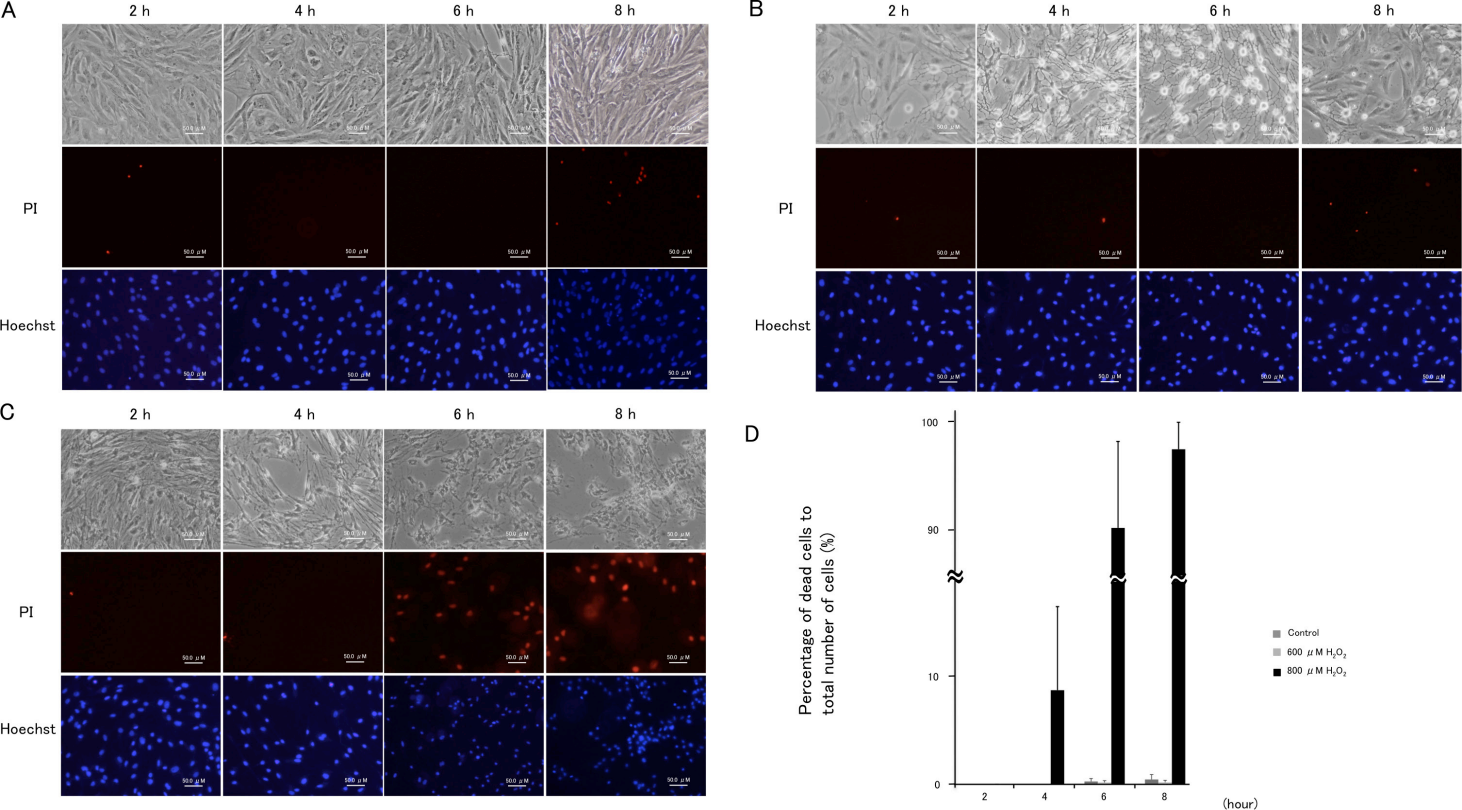
Cellular morphological changes in porcine trabecular meshwork (PTM) cells treated with medium alone, or with 600 μM or 800 μM H_2_O_2_. PTM (1x10^5^) cells were seeded into 12-well plates. Three days later, the medium was changed to serum-free medium overnight, and then PTM cells were treated as followed for various amounts of time: **A**: medium alone, **B**: 600 μM H_2_O_2_ **C**: 800 μM H_2_O_2_. The unfixed cells were stained with propidium iodide to detect cell death (red; middle panel). Nuclei of total cells were stained with Hoechst 33342 (blue; lower panel). The time course for mean percentages of dead cells (propidium iodide-positive cells) to total number of cells (Hoechst 33342-positive cells) is shown (n=3; **D**). Thus, 800 μM H_2_O_2_ increased the number of dead PTM cells for 8 h after treatment, but PTM cells treated with 600 μM H_2_O_2_ recovered from the morphologic changes without significant cell death. Scale bar represents 50 μm.

**Figure 2:**
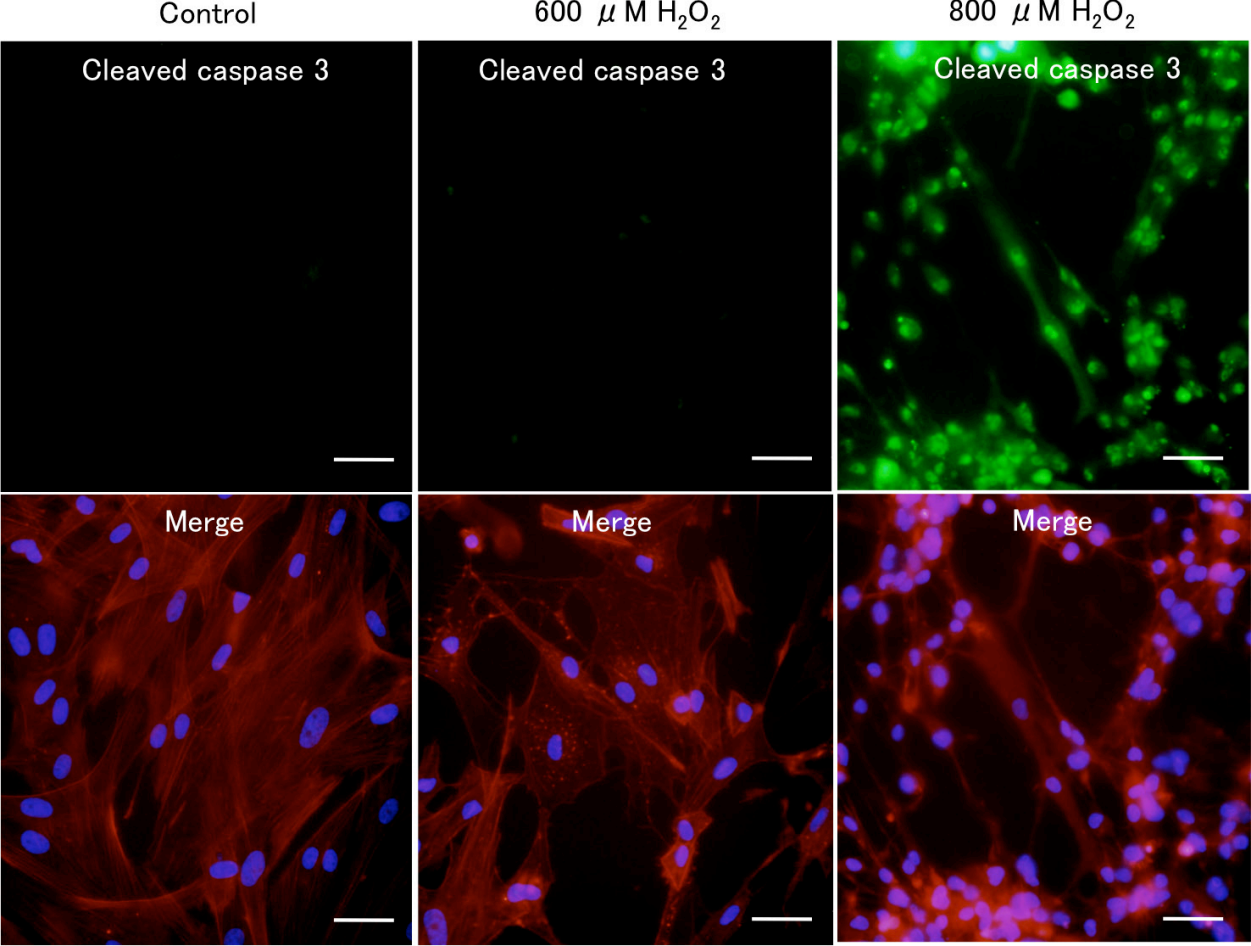
Immunocytochemical staining of H_2_O_2_-treated porcine trabecular meshwork (PTM) cells. PTM cells treated with 600 or 800 μM H_2_O_2_ for 6 h were fixed, permeabilized, and stained with anticleaved caspase-3 antibody (green; upper panels). F-actin was stained with phalloidin-TRITC (red; lower panels). Cell nuclei were counterstained with DAPI (blue). Scale bar represents 50 μm.

**Figure 3:**
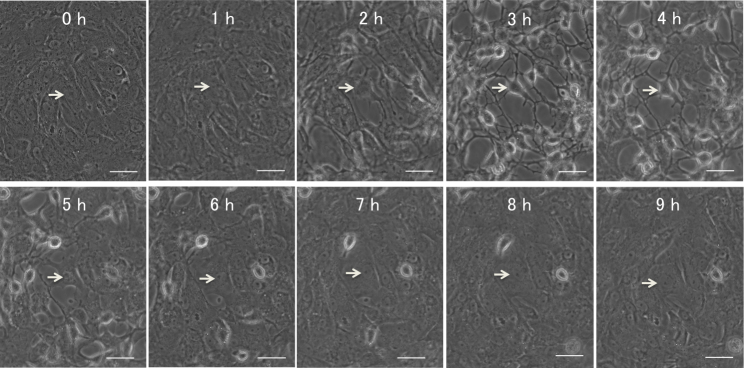
Time-lapse images of porcine trabecular meshwork (PTM) cells treated with 600 μM H_2_O_2_. Time-lapse sequence of PTM cells after treatment with 600 μM H_2_O_2_ were recorded every 15 min. The figure with “0 hour” (left upper) shows PTM cells immediately after H_2_O_2_ treatment. Arrow indicates an affected cell in the sequence. Scale bar represents 50 μm.

Microscopic examination revealed that treatment with 600 μM H_2_O_2_ induced cell–cell separation, rounding up of PTM cells at 2 h was comparable with medium alone using control, and the changed morphology of PTM cells was partially recovered at 8 h (Figure 1A,B). In contrast, PTM cells treated with 800 μM H_2_O_2_ showed similar morphologic changes at 2 h or later with no subsequent recovery (Figure 1C). The percentage of dead cells to total cells, which was estimated by nuclear staining with Hoechst 33342 and PI dyes, was not affected by treatment with 600 μM H_2_O_2_, while treatment with 800 μM H_2_O_2_ increased the percentage of dead cells to 100% at 6 h or later (Figure 1D). Immunocytochemical staining revealed that morphologically changed PTM cells 6 h after treatment with 600 μM H_2_O_2_ were negative for cleaved caspase 3 and presented depolymerized F-actins (Figure 2), suggesting that morphological changes of the PTM cells were accompanied by cytoskeletal rearrangement without apoptosis. In contrast, PTM cells treated with 800 μM H_2_O_2_ were stained by anticleaved caspase-3 antibody, accompanied with condensed nuclei (Figure 2). These data were consistent with the results of PI staining, and indicated 800 μM H_2_O_2_ treatment induced apoptosis in PTM cells, while 600 μM H_2_O_2_ did not. To investigate the time-dependent morphological change of each cell after treatment with 600 μM H_2_O_2_, time-lapse imaging was performed. Over time following treatment with H_2_O_2_, the changed shape of each PTM cell was confirmed as having recovered to the pretreatment shape (Figure 3).

### Identification of oxidative stress response signaling pathways

**Figure 4:**
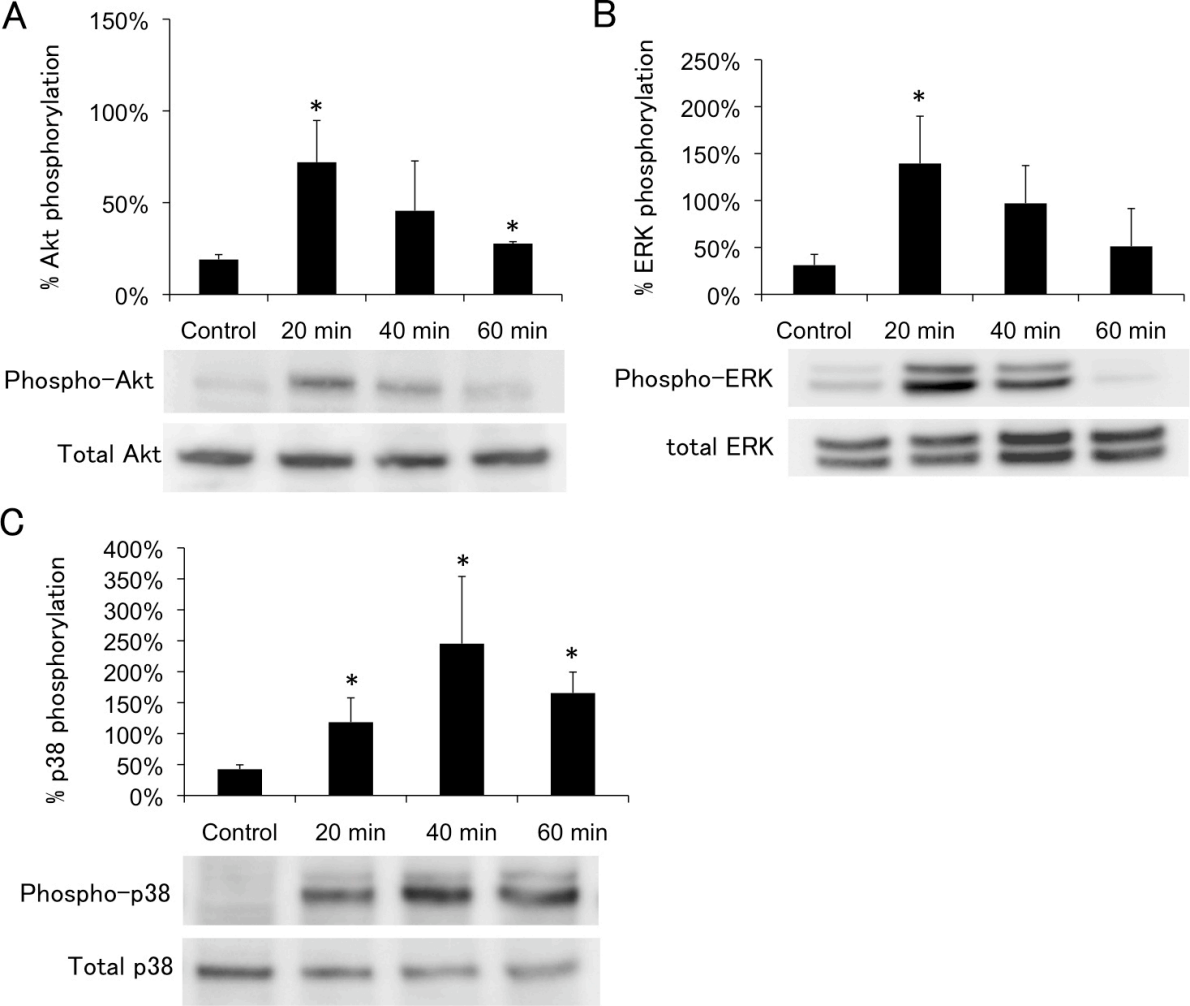
Time course of H_2_O_2_-induced Akt (Ser473), ERK1/2, p38 phosphorylation. Porcine trabecular meshwork (PTM) cells (1x10^5^) in 12-well plates were treated with medium alone (control) or 600 μM H_2_O_2_ for various amounts of time. Time course H2O2-induced Akt (Ser473), ERK1/2, p38 phosphorylation were indicated panels **A**, **B**, and **C**, respectively. Levels of protein bands were quantified, and the ratios of phosphorylated protein to total protein were calculated (n=3). *p<0.05 compared with medium alone. Bars indicate the standard deviations.

**Figure 5:**
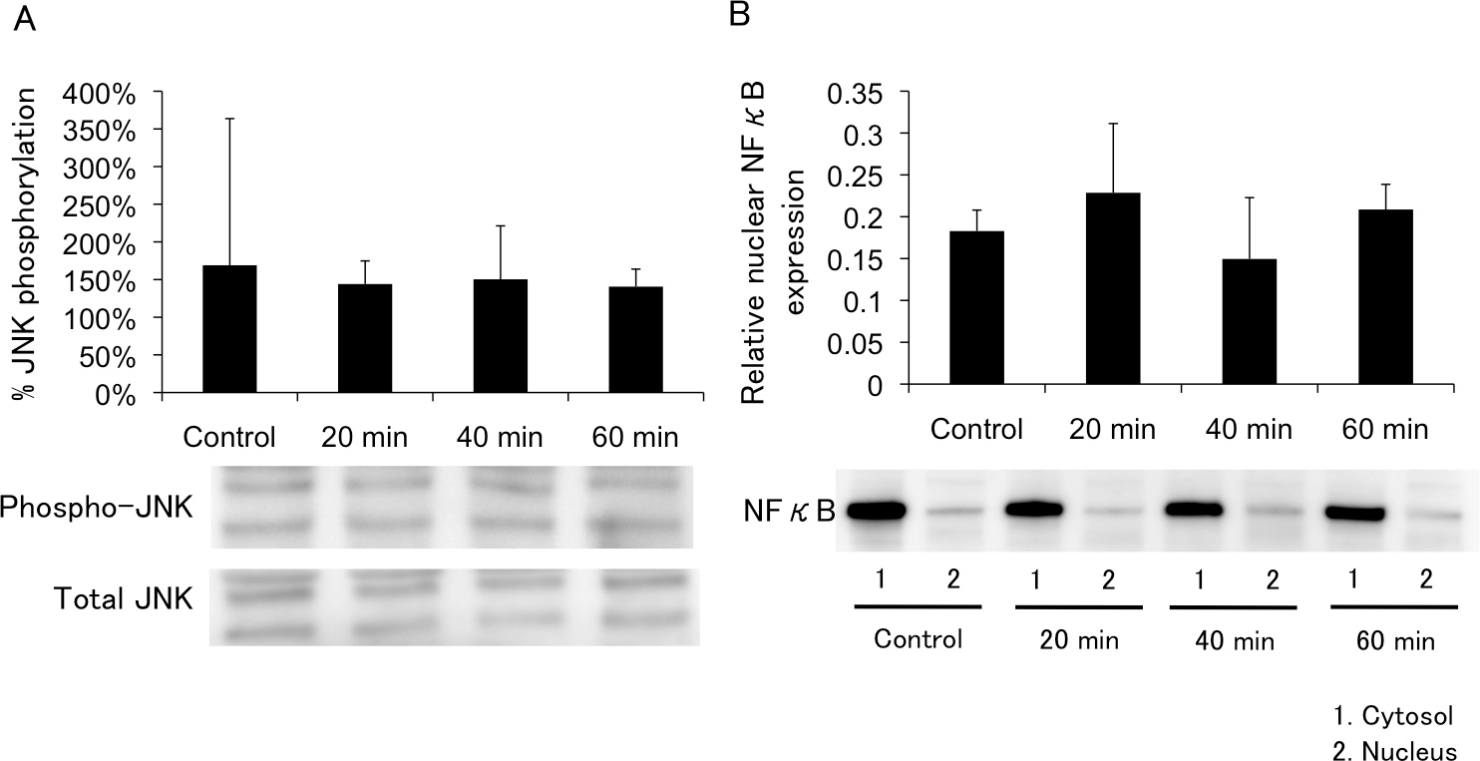
Time course of H_2_O_2_-induced JNK phosphorylation and NFκB. Porcine trabecular meshwork (PTM) cells were treated with medium alone (control) or with 600 μM H_2_O_2_. Cytosol or nucleus fraction was extracted from TM cells. Cytoplasmic proteins were separated by sodium dodecyl sulfate PAGE, transferred onto a polyvinylidene fluoride transfer membrane, and probed with antibody to p-JNK, JNK (**A**), and NFκB (**B**). Levels protein bands were quantified, and the expression ratios of phosphorylated protein to total protein for JNK and nucleus to cytosol for NFκB were calculated (n=3). *p<0.05 compared with medium alone. Bars indicate the standard deviations.

PTM cells were treated with 600 μM H_2_O_2_, and the percentages of phosphorylated signaling molecules were examined by western blotting 20, 40, and 60 min after beginning H_2_O_2_ treatment. Compared with control, the percentage of phosphorylated Akt was significantly increased by 3.8-fold (p=0.016) 20 min after treatment with 600 μM H_2_O_2_, and subsequently declined (Figure 4A). Similarly, the percentage of phosphorylated ERK1/2 also increased to maximum at 20 min (3.6-fold; p=0.021) after treatment with 600 μM H_2_O_2_ (Figure 4B). The percentage of phosphorylated p38 increased for up to 40 min (5.8-fold; p=0.032) after treatment with 600 μM H_2_O_2_ (Figure 4C). The percentage of phosphorylated JNK did not significantly change after treatment with 600 μM H_2_O_2_ (Figure 5A). We also examined the kinetics of the intracellular localization of NFкB after treatment with H_2_O_2_. The expression ratio of nucleus to cytosol represented a 1.3-, 0.8-, and 1.1-fold increase compared with the control, at 20, 40, and 60 min, respectively, after treatment with 600 μM H_2_O_2_. This change in the intracellular localization of NFкB was not statistically significant under any of the conditions examined (Figure 5B). The results of PTM cells treated with 800 μM H_2_O_2_ are not shown, because they were almost dead as time passed.

### Effects of inhibitors of oxidative stress response signaling pathways on cell morphology and cell survival

**Figure 6:**
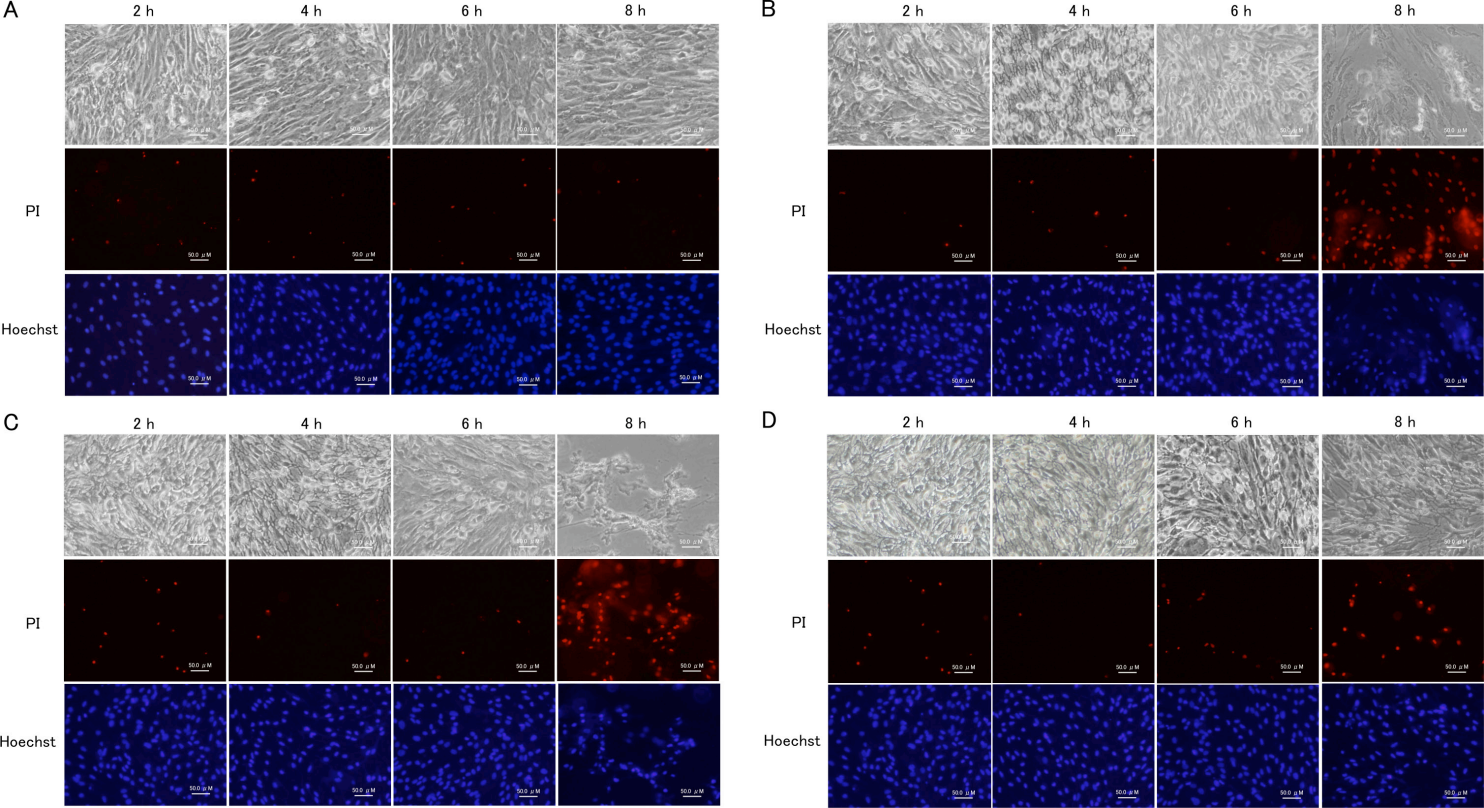
Effects of PI3K-Akt, ERK1/2, p38 blockade in porcine trabecular meshwork (PTM) cells treated with medium alone or 600 μM H_2_O_2_ for various amounts of time. PTM (1x10^5^) cells were seeded into 12-well plates. Three days later, the medium was changed to serum-free medium overnight. PTM cells were pretreated with vehicle (**A**), LY294002 (10 μM; **B**), U0126 (10 μM; **C**), SB203580 (10 μM; **D**), inhibitors of PI3K, ERK1/2, or p38, respectively, for 1 h, and then treated with medium alone (**A**) or 600 μM H_2_O_2_ (**B**–**D**) for various amounts of time. The cells were stained with propidium iodide to detect cell death, (red; middle panel). Nuclei were stained with Hoechst 33342 (blue; lower panel).

**Figure 7:**
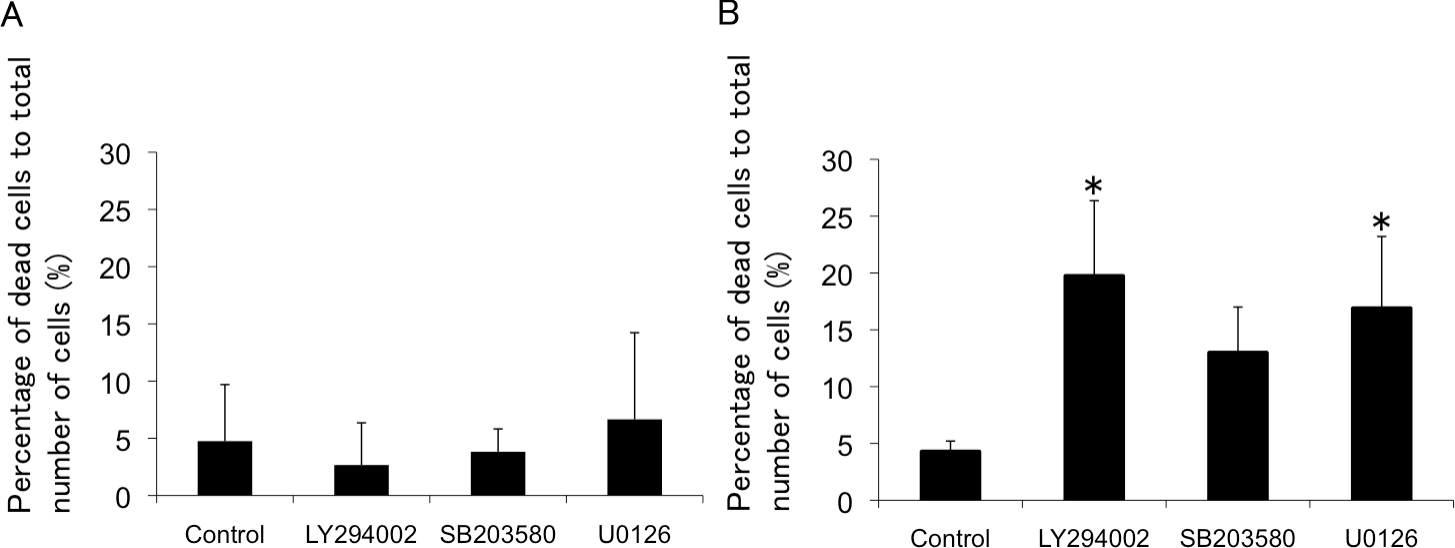
Sensitivity of porcine trabecular meshwork (PTM) cells to H_2_O_2_ for 8 h after pretreatment with LY294002, U0126, and SB203580. The mean percentages of dead cells (propidium iodide-positive cells) to total number of cells (Hoechst 33342-positive cells) were estimated with medium alone (**A**) or 600 μM H_2_O_2_ (**B**) treatment for 8 h after pretreatment with each inhibitor or vehicle (n=4). *p<0.05 compared with control. Bars indicate the standard deviations.

PTM cells were pretreated with LY294002, U0126, or SB203580, and with inhibitors of PI3K, ERK1/2, or p38, respectively, and the H_2_O_2_-induced morphologic changes were investigated. Following pretreatment with an inhibitor of PI3K, an upstream molecule of Akt [17], H_2_O_2_-induced morphologic changes, such as cell–cell separation and rounding up of cells, were observed as early as 2 h, and did not recover even at 8 h after 600 μM H_2_O_2_ treatment (Figure 6B). The dead cells (PI-positive cells) were increased at 8 h after 600 μM H_2_O_2_ treatment. Pretreatment with an Akt inhibitor induced similar changes as the PI3K inhibitor (data not shown). In the presence of an ERK1/2 inhibitor, similar morphologic changes were observed after treatment with 600 μM H_2_O_2_. The dead cells were increased at 8 h after 600 μM H_2_O_2_ treatment (Figure 6C). The effect of pretreatment with a p38 inhibitor was comparable to that with the ERK1/2 inhibitor, but less effective (Figure 6D). Statistical analysis showed the ratios of PI-positive cells to Hoechst 33342-positive cells were not different among the groups without H_2_O_2_ treatment, suggesting the inhibitors themselves did not induce cell death. In contrast, the ratio significantly increased after 600 μM H_2_O_2_ treatment in the presence of LY294002 (p=0.007) or U0126 (p=0.023), compared with vehicle-treated cells. The ratio also tended to increase in the presence of SB203580, but the difference was not significant (p=0.120; Figure 7).

## Discussion

In the present study, we demonstrated that H_2_O_2_-induced oxidative stress changed cell morphology, which was reversible in part, and activated intracellular signaling pathways of PI3K-Akt, ERK1/2, and p38 in PTM cells at 20 min. Furthermore, when either PI3K-Akt or ERK1/2 was inhibited, H_2_O_2_-treated PTM cells showed irreversible morphologic changes and increased cell death. We also demonstrated that morphological changes of H_2_O_2_-treated PTM cells were accompanied by depolymerized F-actin, without apoptosis. Though data produced without genetic modification may provide a limited explanation of signal transduction mechanisms, the results of the present study are the first to clarify the primary oxidative stress response signaling pathways in TM cells and their effects on cell viability.

Some investigators have demonstrated that oxidative stress is involved in glaucoma pathology [12,13,18], and that the trabecular meshwork is the tissue most sensitive to oxidative radicals in the anterior chamber [19]. In the present study, PTM cells showed morphologic changes 2 h after treatment with 600 or 800 μM H_2_O_2_. In other ocular cells, similar concentrations of H_2_O_2_ also induced cytotoxic effects [16,20]. In human TM cells, 600 and 800 μM H_2_O_2_ increases the number of dead cells [20]. In PTM cells in the present study, 800 μM H_2_O_2_, but not vehicle alone or 600 μM H_2_O_2_, increased the number of dead cells for 8 h after treatment. This shows that PTM cells may have better tolerance against oxidative stress than human TM cells in the past report [20]. In the present study, PTM cells treated with 600 μM H_2_O_2_ recovered from the morphologic changes without significant cell death. These findings suggest that PTM cells have some antioxidative mechanisms and overcome relatively slight oxidative stress, even after undergoing some morphologic changes. Interestingly, the morphologic changes were accompanied by depolymerization of F-actin in the present study. Although it was reported that Nox1-induced reactive oxygen species inactivated Rho in rat kidney cells [21], the molecular mechanism in TM cells remains elusive.

Oxidative stress induces activation of the PI3K-Akt signaling pathway in other cell types [17,22-24]. In PTM cells treated with 600 μM H_2_O_2_, Akt phosphorylation was significantly increased at 20 min and then decreased in a time-dependent manner. In human retinal pigment epithelial cells treated with 200 μM H_2_O_2_, phospho-Akt levels increased to a maximum at 15 min and decreased slowly, and pretreatment with LY294002 for 1 h reduced H_2_O_2_-induced Akt phosphorylation in retinal pigment epithelial cells nearly to the basal level [22]. Similarly, we showed that inhibition of PI3K-Akt signaling by LY294002, with cell–cell separation and rounding up, suppressed the recovery of PTM cells from the H_2_O_2_-induced abnormal morphology. These findings indicate that oxidative stress activates the PI3K-Akt signaling pathway in TM cells, and that the signaling is involved in the mechanism of recovery from abnormal morphologic changes.

It is widely accepted that ERK1/2 activation is required for cell survival [25,26]. In PTM cells treated with 600 μM H_2_O_2_, ERK1/2 phosphorylation was significantly increased at 20 min and then decreased time-dependently. In rat-lens epithelial cells treated with H_2_O_2_, the first peak of the phospho-ERK 1/2 level reached a maximum at 15 min, followed by a decline toward baseline within 30 min [27]. In other cell types as well, oxidative stress induces increases in the phospho-ERK 1/2 level [24,28]. Together, these data indicate that acute oxidative stress induces activation of ERK1/2 in several ocular cell types.

In the present study, p38 phosphorylation was increased time-dependently, reached a maximum at 40 min, and then decreased time-dependently in PTM cells treated with H_2_O_2_. In studies of other cell types, phospho-p38 levels increased to a maximum at 1 h, and then decreased time-dependently [29]. In the present study, inhibition of p38 affected cell morphology under oxidative stress, indicating that p38 signaling occurs in response to oxidative stress and has some role in the maintenance of cell morphology in TM cells. Pretreatment with a p38 inhibitor, however, did not significantly affect cell viability under oxidative stress conditions, compared to vehicle-treated control. Thus, the effects of p38 on cell viability require further investigation.

JNK is a MAPK family member that reacts to various stresses, such as UV light and oxidative stress [30,31]. To confirm that JNK activation is induced by oxidative stress, we examined the level of JNK activation caused by treatment with H_2_O_2_. Our data revealed that JNK was not activated in PTM cells until 1 h after treatment with H_2_O_2_. H_2_O_2_-induced oxidative stress induces activation of JNK in other cell types [27,29,32]. In previous studies, H_2_O_2_-induced JNK activation started between 5 min and more than 1 h. Similarly, H_2_O_2_-induced NFкB activation was not detected in PTM cells until 1 h after treatment with both doses of H_2_O_2_. NFкB is an important nuclear transcription factor that mediates an inflammatory response caused by oxidative stress [33]. In endothelial cells, NFкB is activated strongly by treatment with H_2_O_2_ for 4 h [34]. In PTM cells, H_2_O_2_-induced activation of JNK or NFκB might continue beyond the stimulation time. Thus, longer treatment with H_2_O_2_ might activate JNK and NFкB signaling pathways in TM cells. Even if this is the case, however, JNK and NFкB signaling pathways may not be primary reactive pathways.

What is the clinical implication of the present study? TM cells have important roles, such as maintenance of the ECM and phagocytosis of the debris in outflow tissues, to control aqueous-outflow facility. Thus, a shortage of TM cells might result in a failure of control, leading to decreased outflow facility and subsequent ocular hypertension. In this context, oxidative stress is a potential factor in reducing the number of TM cells, and might thus be involved in glaucoma pathology. Elucidation of the primary oxidative stress response signaling pathways and their roles in cell viability might reveal promising targets for protecting TM cells from oxidative stress, and thereby contribute to preventing blindness from glaucoma.

In conclusion, PI3K-Akt, ERK, and p38 signaling pathways are primary oxidative stress response pathways in TM cells, shown to be involved in the mechanism of recovery from cellular morphologic changes after H_2_O_2_ treatment, accompanied by actin cytoskeletal changes.
